# OUTDOOR ENVIRONMENTAL RISK FACTORS FOR FALLS AND FEAR OF FALLING: A SYSTEMATIC REVIEW

**DOI:** 10.1093/geroni/igac059.1014

**Published:** 2022-12-20

**Authors:** Pamela Dunlap, Zachary Hubbard, Erica Fan, Elsa Strotmeyer, Andrea Rosso

**Affiliations:** University of Pittsburgh, Pittsburgh, Pennsylvania, United States; University of pittsburgh Geriatric Psychiatry Neuroimaging Program, Pittsburgh, Pennsylvania, United States; University of Pittsburgh, Pittsburgh, Pennsylvania, United States; School of Public Health, University of Pittsburgh, Pittsburgh, Pennsylvania, United States; University of Pittsburgh, Pittsburgh, Pennsylvania, United States

## Abstract

The purpose of this systematic review was to identify outdoor environmental risk factors associated with falls and/or fear of falling among older adults. PubMed, EMBASE, and CINAHL were searched through February 19, 2021. Studies were included if they measured outdoor environment, falls or fear of falling as an outcome, and included adults aged ≥ 45 years. Excluded studies were not published in English and/or did not include original results. Two study-team members completed abstract screening. For full text reviews, a third reviewer resolved conflicts. A total of 5,727 abstracts were screened and 462 full texts were reviewed. After full-text review, approximately 25 studies will be assessed for risk of bias and data extracted by two independent reviewers. Based on the initial review, uneven outdoor surfaces, busy traffic areas, and neighborhood disorder were associated with falls/fear of falling among older adults. Modifiable outdoor environmental factors may be targets for fall prevention.

